# Parameters associated with successful weaning of veno-arterial extracorporeal membrane oxygenation: a systematic review

**DOI:** 10.1186/s13054-022-04249-w

**Published:** 2022-12-05

**Authors:** Francis Charbonneau, Karina Chahinian, Emmanuel Bebawi, Olivier Lavigueur, Émilie Lévesque, Yoan Lamarche, Karim Serri, Martin Albert, Pierre-Emmanuel Noly, Alexis Cournoyer, Yiorgos Alexandros Cavayas

**Affiliations:** 1 Division of Critical Care, Department of Medicine, Hôpital du Sacré-Cœur de Montréal, 5400 Boulevard Gouin Ouest, Montreal, QC H4J 1C5 Canada; 2 Division of Critical Care, Department of Surgery, Montreal Heart Institute, Montreal, Canada; 3 Department of Emergency Medicine, Hôpital du Sacré-Cœur de Montréal, Montreal, Canada

**Keywords:** Cardiogenic shock, Extracorporeal membrane oxygenation, Extracorporeal life support, VA-ECMO, Adults, Biomarkers, Left ventricular function, Right ventricular function, Echocardiography, Hemodynamic parameters

## Abstract

**Purpose:**

Veno-arterial (VA) extracorporeal membrane oxygenation (ECMO) can be used to restore organ perfusion in patients with cardiogenic shock until native heart recovery occurs. It may be challenging, however, to determine when patients can be weaned successfully from ECMO—surviving without requiring further mechanical support or heart transplant. We aimed to systematically review the medical literature to determine the biomarkers, hemodynamic and echocardiographic parameters associated with successful weaning of VA-ECMO in adults with cardiogenic shock and to present an evidence-based weaning algorithm incorporating key findings.

**Method:**

We systematically searched PubMed, Embase, ProQuest, Google Scholars, Web of Science and the Grey literature for pertinent original research reports. We excluded studies limited to extracorporeal cardiopulmonary resuscitation (ECPR) as the neurological prognosis may significantly alter the decision-making process surrounding the device removal in this patient population. Studies with a mixed population of VA-ECMO for cardiogenic shock or cardiac arrest were included. We excluded studies limited to patients in which ECMO was only used as a bridge to VAD or heart transplant, as such patients are, by definition, never “successfully weaned.” We used the Risk of Bias Assessment tool for Non-Randomized Studies. The study was registered on the International prospective register of systematic reviews (PROSPERO CRD42020178641).

**Results:**

We screened 14,578 records and included 47 that met our pre-specified criteria. Signs of lower initial severity of shock and myocardial injury, early recovery of systemic perfusion, left and right ventricular recovery, hemodynamic and echocardiographic stability during flow reduction trial and/or pump-controlled retrograde trial off predicted successful weaning. The most widely used parameter was the left ventricular outflow tract velocity time integral, an indicator of stroke volume. Most studies had a moderate or high risk of bias. Heterogeneity in methods, timing, and conditions of measurements precluded any meta-analysis.

**Conclusions:**

In adult patients on VA-ECMO for cardiogenic shock, multiple biomarkers, hemodynamic and echocardiographic parameters may be used to track resolution of systemic hypoperfusion and myocardial recovery in order to identify patients that can be successfully weaned.

**Graphical Abstract:**

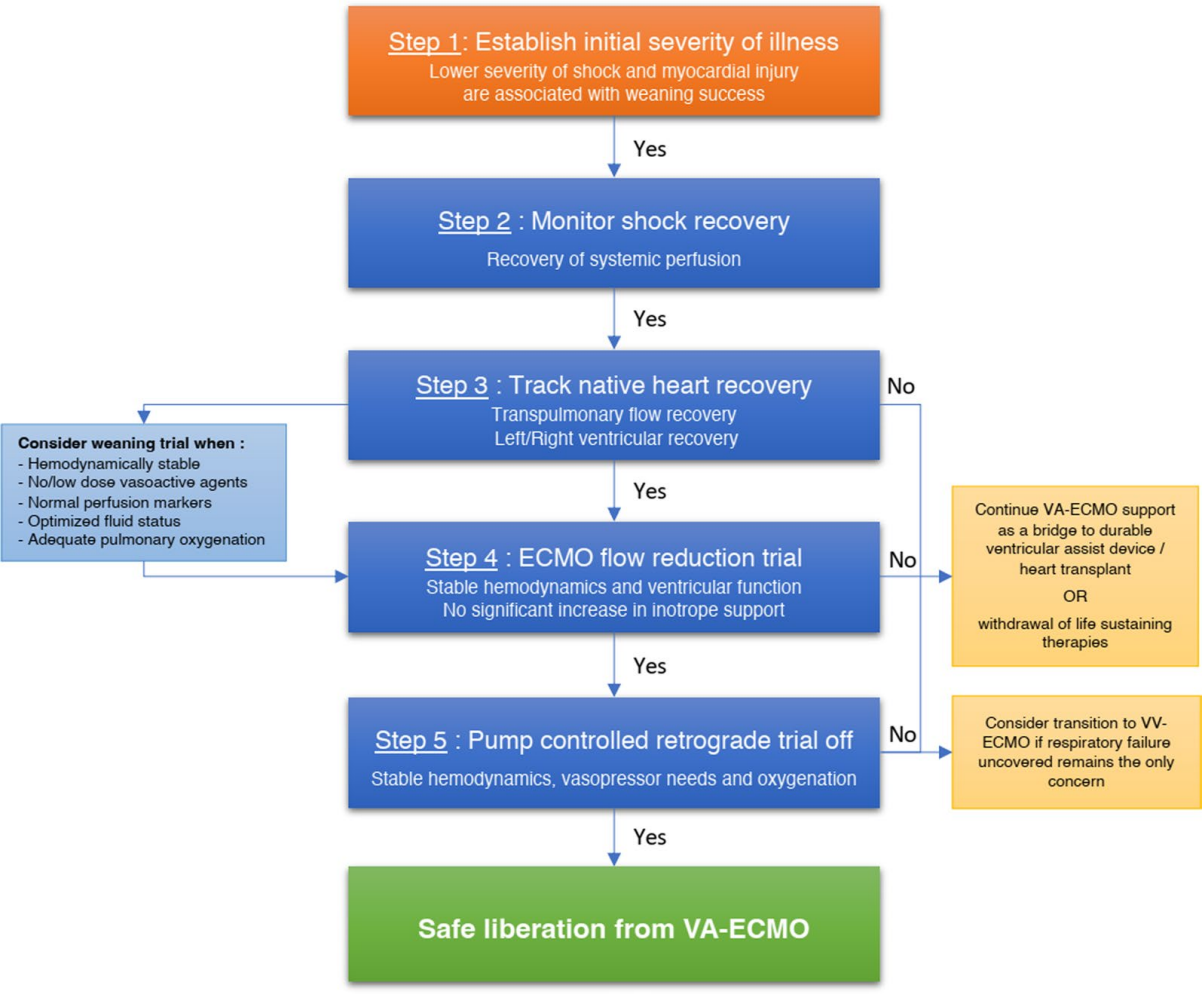

**Supplementary Information:**

The online version contains supplementary material available at 10.1186/s13054-022-04249-w.

## Take-home message

In adult patients on VA-ECMO for cardiogenic shock, the following indices predicted successful weaning from VA-ECMO (survival after removal of ECMO without requirement for further mechanical support or heart transplant):

- Lower severity of initial shock (MAP, lactates) and myocardial injury (Troponins).
- Early recovery of systemic perfusion (lactate, liver enzymes, microcirculation).
- Left ventricular recovery (LVEF > 20–25%, LVOT VTI > 10 cm, Mitral TDSa > 10 cm/s).
- Right ventricular recovery (TAPSE ≥ 19 mm, RVEF ≥ 25%, Low RA/PCWP, High PAPi).
- Stable MAP, CI, SBP without significant increase in inotropic support and stable or improved LV/RV function during flow reduction trial and PCRTO.

Most studies were observational, unblinded, retrospective and had small sample size and a moderate or high risk of bias.

## Introduction

Veno-arterial (VA) extracorporeal membrane oxygenation (ECMO) can be used to restore organ perfusion in patients with cardiogenic shock [1, 2]. The device drains deoxygenated blood from a venous inflow cannula, drives it through a membrane lung and returns oxygenated blood in an arterial outflow cannula, providing both respiratory and cardiac support [3]. VA-ECMO can be used as a bridge to recovery, restoring and maintaining systemic perfusion while cardiac recovery occurs. In the absence of myocardial recovery, VA-ECMO may act as a bridge to durable ventricular assist device (VAD) implantation or heart transplant [4]. Successful weaning of VA-ECMO is generally defined as survival after complete removal of the extracorporeal circuit without requirement for further mechanical support or heart transplant [5–8]. Reported success rates range from 30 to 75% [5–9]. The timing of weaning is crucial as premature withdrawal may lead to recurrence of shock and cause secondary injury on barely recuperating organs. Conversely, longer duration of ECMO is associated with higher complications and in-hospital mortality [10, 11]. Identifying patients who are ready to be weaned off VA-ECMO may be challenging. The Extracorporeal Life Support Organization (ELSO) recommends that weaning be attempted in hemodynamically stable patients on low vasoactive support with the use of echocardiography to assess myocardial recovery [12–14]. Echocardiographic indices [15] are widely used to assess readiness to be weaned. Other parameters, such as biomarkers [16] or hemodynamic parameters [17] may also be used. Despite numerous descriptions of weaning protocols based on expert opinion in the literature [7, 18, 19], there has been no systematic reviews to provide more robust guidance. We aimed to systematically review the medical literature to determine the biomarkers, hemodynamic and echocardiographic parameters associated with successful weaning of VA-ECMO in adults with cardiogenic shock. Secondarily, we aimed to present an evidence-based weaning algorithm incorporating key findings to guide clinicians in the weaning process.

## Methods

The protocol for this systematic review was registered on the International Prospective Register of Systematic Reviews (CRD42020178641). The results are reported according to the Preferred Reporting Items for Systematic Reviews and Meta-Analysis Guidelines [20].

### Study characteristics

**Fig. 1:**
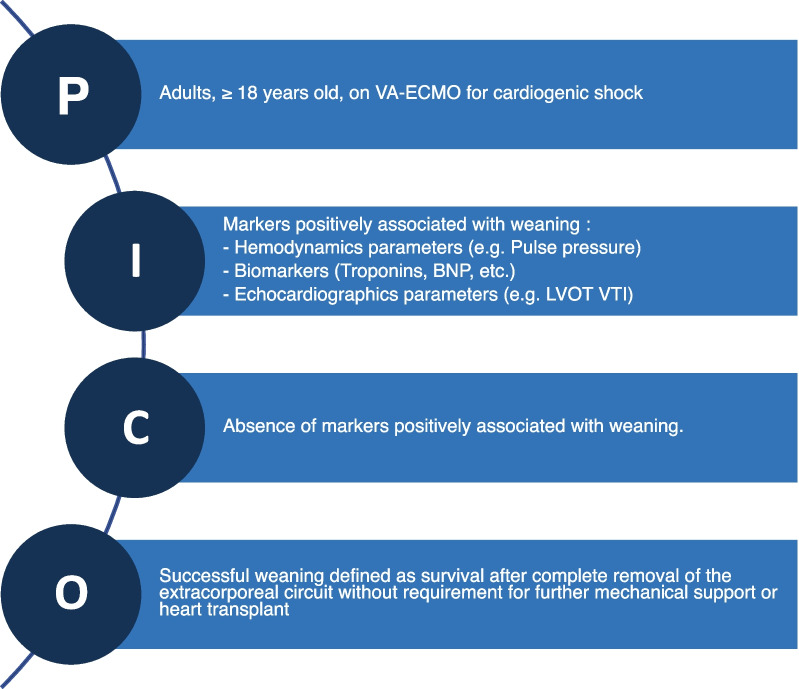
Search strategy. *NP* brain natriuretic peptide, *C* comparison, *I* intervention, *LVOT VTI* left ventricular outflow tract velocity–time integral, *O* outcome, *P* population

As we did not expect randomized controlled trials to be available on the subject, we elected to include both interventional and observational studies, including cohort studies and case-series ≥ 10 patients. We included records in all languages, both in full text or abstract-only formats, published from database inception to April 10, 2022. Studies had to report on the association between biomarkers, hemodynamic and echocardiographic parameters and VA-ECMO weaning success. Studies evaluating the association between therapies and weaning success were excluded. Studies evaluating exclusively the association of baseline parameters (before ECMO initiation) and weaning success were excluded. The PICO strategy is detailed in Fig. 1.

### Participants

We selected studies that included adults with cardiogenic shock secondary to potentially reversible etiologies treated with VA-ECMO. We excluded studies limited to extracorporeal cardiopulmonary resuscitation (ECPR) as the neurological prognosis may significantly alter the decision-making process surrounding the device removal in this patient population. Studies with a mixed population of VA-ECMO for cardiogenic shock or cardiac arrest were included. We excluded studies limited to patients in which ECMO was only used as a bridge to VAD or heart transplant, as such patients are, by definition, never “successfully weaned.”

### Search strategy

The search strategy is detailed in Additional file 1: Table S2. We searched Medline and Embase for studies including the following concepts: VA-ECMO, ECMO, extracorporeal life support (ECLS), ECPR, cardiogenic shock, weaning, success, decannulation. ProQuest, Google Scholar and Grey literature (OpenGrey, GreyLit, GreyNet) were also searched using key search terms. Backward and forward citation tracking was performed using Web of Science. Conference abstracts indexed in Embase and these other sources were eligible for inclusion. The searches were rerun prior to the final analyses (April 10, 2022) and further studies retrieved for inclusion.

### Study selection process

Covidence systematic review software (Veritas Health Innovation, Melbourne, Australia) was used for the selection process. Two independent members of the review team (FC and EB or OL or KC) first screened the citations using only titles and abstracts and assessed the full texts for eligibility. Conflicts were resolved by consensus by the corresponding author (YAC).

### Data extraction and synthesis

Study design, setting, country, period, sample size, funding source, VA-ECMO indication, weaning protocol, successful weaning definition and reported value, biomarkers, hemodynamic and echocardiographic data as well as effect measures between exposition and outcome were extracted independently by authors on electronic data collection forms (Covidence software). Missing data were presented as not reported in Table 1. The primary outcome was weaning success, defined as survival after complete removal of the extracorporeal circuit without requirement for further mechanical support or heart transplant. We planned to present a narrative synthesis of findings and a meta-analysis of the diagnostic accuracy of parameters to predict a successful weaning if they had been studied by multiple groups under similar conditions.

**Table 1:** Characteristics of included studies

References	Country; study period	Design; setting; sample size	Indication for VA-ECMO (%)	Weaning protocol	Parameters measured: (B) biomarkers, (H) hemodynamic, (E) echocardiographic	Successful weaning definition	Weaning success (%) (as reported by authors)
Aissaoui et al. 2011 [15]	France; 2007–2008	Prospective cohort; single center; n=38	CMP (47%) FM (6%) Post-cardiotomy shock (22%) Post-transplantation (10%) Other (16%)	66% flow (15 min) 33% flow or 1–1.5 L/min (15 min) Return to 100% if unstable any level If stable minimal flow, VTI > 10 cm, LVEF > 20–25%, circuit clamp and decannulation	(B) ABG, Lact, Creat; (H) MAP, SBP/DBP, PAP; (E) LVEF, LVOT VTI, TD E, Sa, Ea, E/Ea, RA/RV size	ECMO removal and no further MCS because of recurring CS over the following 30 days	20/38 = 53%
Aissaoui et al. 2012 [52]	France; 2007	Prospective cohort; single center; n=22	CMP (50%) Post-cardiotomy shock (32%) FM (4.5%) Post-transplantation (4.5%) Other (9%)	See Aissaoui 2011	(B) N/A; (H) MAP, SBP, DBP, HR; (E) LVOT VTI, LVEDV, LVEF, TD E VVI: TD Sa, Ea, Sv, strain, strain rate	ECMO removal and no further MCS because of recurring CS over the following 30 days	11/22 = 50%
Aissaoui et al. 2017 [55]	France; 2007–2008	Prospective study; single center; n=33	See Aissaoui 2012	See Aissaoui 2011	(B) Creat, pH, Lact; (H) N/A; (E) LV/RV size, LVEF, LVOT VTI, TD E, Ea, Sa, RVEF, MR, TR	ECMO removal and no further MCS because of recurring CS over the following 30 days	16/33 = 48%
Akin et al. [45]	Netherlands; 2014–2016	Prospective cohort; single center; n=13	PE (38%) Post-cardiotomy shock (23%) CS post-AMI (15%) Myocarditis (15%) Intoxication (8%)	50% flow If stable, VTI > 10 cm, LVEF > 20–25% at minimal flow, decannulation	(B) Lact, S/L circulation (TVD, PVD, PPV); (H) MAP; (E) LVOT VTI, LVEF, TAPSE, TDSa	Successful VA-ECMO explantation within 48 h	10/13 = 77%
Asaumi et al. [29]	Japan; 1993–2001	Retrospective cohort; single center; n=14	Fulminant myocarditis (100%)	LVETc improved to > 200 ms, ECMO flow rate decreased until 1.5 L/min If stable, decannulation	(B) CK, CK-MB, WBC, CRP, AST/ALT, Creat, BUN; (H) CI, PCWP, RAP; (E) LVETc, LVESD, LVEDD, LVWT, FS, MR, TR	ECMO removal	10/14 = 71%
Cavarocchi et al. [7]	USA; 2011–2012	Prospective cohort; single center; n=21	CMP (48%) Myocarditis (14%) CS post-AMI (14%) Post-cardiotomy (10%) PE (5%)	50% flow by 0.5 L/min decrement Volume challenge Minimal flow: 1–1.5 L/min Return 100% if distension any level Decannulation if adequate Biventricular function	(B) N/A; (H) N/A; (E) Qualitative (hTEE): LV/RV function and size, LV FAC	ECMO removal	14/21 = 67%
Chen et al. [5]	Taiwan; NR	Retrospective cohort; single center; n=57	NR	Gradual flow reduction Return to 100% if inotrope increase above predefined dose If stable, decannulation	(B) CK, CK-MB, Troponins, Bun, Creat, AST, RNI, CBC; (H) N/A; (E) N/A	Weaning from ECMO and survival beyond 48 h	38/57 = 67%
Chommeloux et al. [44]	France; NR	Prospective cohort; single center; n=14	CS post-AMI (50%) CMP (28%) Graft failure (14%) FM (7%)	NR	(B) Lact, S/L circulation: SVD, PSVD, PPV, MFI, HI; (H) MAP, HR; (E) LVEF, VTI	ECMO removal	6/14 = 43%
Colombo et al. [22]	Italy; 2013–2017	Retrospective cohort; single center; n=25	CPR (71%) CS post-AMI (17%) Myocarditis (7%) PE (4%) Tako-tsubo (2%) Intoxication (2%)	First weaning trial at 48 h no additional details	(B) N/A; (H) SV, CO; (E) LV t-IVT, LVEF, LVEDD, MAPSE	Device removal without requirement for re-cannulation over the following 30 days	18/25 = 72%
Elena Puerto et al. [59]	Spain; NR	Retrospective cohort; NR; n=87	NR	NR	(B) N/A; (H) N/A; (E) RV dysfunction, RV basal diameter	NR	NR
Frederiksen et al. [56]	Denmark; NR	Cohort; single center; n=15	NR	Stable and VTI > 7 cm no additional details	(B) N/A; (H) N/A; (E) LVOT VTI, LVEF, TD S', TAPSE	ECMO weaning and being alive 24 h later without hemodynamic MCS	15/29 = 52%
Fried et al. [37]	USA; 2008–2018	Retrospective cohort; Single center; n=126	CS post-AMI (100%)	Daily flow reduction to 1L/min once on low-dose inotrope. Hemodynamic and echocardiographic follow up to decide decannulation	(B) Lactates, Create, peak CK; (H) MAP; (E) LVEF	Ventricular recovery defined as survival to discharge without durable LVAD or HT	39/126 = 31%
Gambaro et al. [57]	Italy; NR	Prospective cohort; single center; n=14	NR	NR	(B) N/A; (H) HR, MAP, CO, SV; (E) CSt/LS (LV, by STE) LVEF, LVOT VTI	ECMO weaning without adverse outcome within 1 year (MCS, transplant, CV death)	N/A
Gonzalez Martin et al. [27]	Spain; 2013–2020	Cohort; Single center; n=85	CS (47%) ECPR (9%) Electrical storm (9%) Post-cardiotomy CS (33%) Other (1%)	NR	(B) N/A; (H) N/A; (E) LVEDD, LVEF, LVOT VTO, RV basal diameter, RV qualitative function, 1:1 aortic valve aperture	Survival > 24 h after explant and no mortality from cardiogenic shock/heart failure or cardiac arrest during admission	52/85 = 61%
Hsu et al. [39]	Taiwan; NR	Cohort; single center; n=133	NR	Flow reduction trial (< 1.5L/min) If tolerated, ECMO removal	(B) ABG, Lact, Bic; (H) SBP/DBP, CVP, SVO_2_; (E) LVEF	ECMO removal and survival to discharge	73/133 = 55%
Huang et al. [61]	Taiwan; 2014–2015	Retrospective cohort; single center; n=46	NR	Weaning trial when stable Flow reduction to 0.5 L/min (5 min) If tolerated, circuit clamp and decannulation	(B) N/A; (H) HR, CVP, SV (LV/RV); (E) LVEF, CSt/LS (LV), LV size, MR, RVEF, RV FAC, GLS (RV), RV size, TAPSE, TR	ECMO removal and no mortality and/or MCS because of recurring CD over the following 48 h	28/46 = 61%
Joseph et al. [49]	USA; NR	Retrospective cohort; single center; n=30	NR	NR	(B) N/A; (H) RA/PCWP, TPG, PAPi; (E) LVEF, LVEDD, FS	NR	NR
Kim et al. 2021 (JASE) [58]	South Korea; 2016–2018	Prospective cohort; multicenter; n=92	CS post-AMI (48%) Ischemic cardiomyopathy	30–50% flow (15 min) If unstable back to previous flow	(B) N/A; (H) N/A; (E) LVEF, Mitral E/A, Mitral TDI (S' e' a'), LVOT VTI, RVFAC, TAPSE, Tricuspid TDI (S')	ECMO removal and not requiring further MCS over the following 30 days	64/92 = 70%
Kim et al. 2021 (JACC-imaging) [60]	South Korea; 2016–2019	Prospective cohort; single center; n=79	Post-MI CMP (52%) Idiopathic dilated CMP (18%) Fulminant myocarditis (4%) Stress-induced CMP (4%)	If HD stable with low/no vasopressor support, MAP ≥ 65 mmHg, lactate < 2 mmol/L, CVP ≤ 15 mmHg, then gradual weaning	(B) N/A; (H) N/A; (E) Tricuspid annular S′/RVSP RVFAC/RVSP TAPSE/RVSP [RV FWLS]/RVSP	Successful removal of VA-ECMO and no further mechanical circulatory support in the following 30 days	50/79 = 63%
Li et al. [16]	China; 2011–2012	Retrospective cohort; single center; n=123	Post-cardiotomy shock	Gradual flow reduction to 1 L/min If stable, decannulation	(B) Lact, Lact clearance; (H) N/A; (E) N/A	ECMO removal and no HD deterioration within 48 h after	69/123 = 56%
Lim et al. [48]	South Korea; 2010–2018	Cohort; NR; n=122	NR	NR	(B) N/A; (H) HR, MAP, PP; (E) LVEF, LVOT VTI, TDSa	NR	72/122 = 59%
Ling et al. [63]	China; 2010	Observational study; single center; n=30	Post-cardiotomy shock (57%) Myocarditis (14%) CMP (29%)	Reduce speed to target retrograde flow of 0.5–1 L/min and Sweep gas off (1 h) If tolerated, decannulation	(B) N/A; (H) PCRTO; (E) N/A	N/A	7/7 decannulated = 100%
Luyt et al. [23]	France; 2009–2010	Prospective cohort; single center; n=41	CS post-AMI (27%) Myocarditis (17%) Post-cardiotomy (15%) Graft failure (17%) Septic shock (10%) CPR (7%) Rhythm disturbance (7%)	66% flow (15 min) 33% flow or minimum of 1–1.5 L/min (15 min) If unstable, return to 100% flow If stable minimal flow, LVEF > 20–25%, VTI > 12 cm, Mitral systolic velocity > 6 cm Decannulation	(B) NT-proBNP, MR-proANP, proADM, Copeptin, TNIc; (H) N/A; (E) N/A	ECMO removal and survival without MCS for > 30 days	18/41 = 44%
Matsumoto et al. [30]	Japan; 1995–2014	Retrospective cohort; single center; n=37	Myocarditis (100%)	Weaning trial when LVETc > 200 ms Gradual flow reduction to 1.5 L/min If stable, decannulation	(B) CK, CK-MB, ABG, Lact, Bun, Creat, Bili; (H) HR, MAP; (E) LVEF, LV size, LVPWT	ECMO removal	22/37 = 59%
Mazet et al. [24]	France; 2014–2016	Cohort; single center; n=31	CS (71%) CPR (29%)	Gradual decrease to < 2 L/min (60 min) If stable, decannulation	(B) Lact; (H) N/A; (E) LVEF	NR	NR
Mongkolpun et al. [36]	Belgium; NR	Cohort; NR; n=22	CS post-AMI (64%) Post-cardiotomy (14%) Myocarditis (14%) PE (8%)	Gradual flow 1 L/min If VTI > 10 cm, decannulation	(B) Lact, SBF; (H) MAP, CI, SVO_2_; (E) N/A	ECMO removal and HD Stabilization without the need to increase the vasopressor dose within 24 h	12/22 = 55%
Morisawa et al. [38]	Japan; 2006–2008	Retrospective cohort; single center; n=29	CS post-AMI (100%)	NR	(B) BE; (H) HR, Peak BP, SVO_2_; (E) LVEF	ECMO removal and survival for more than one month	15/29 = 52%
Mork et al. [25]	Denmark; 2017–2019	Prospective cohort; single center; n=38	CPR (61%) Heart failure (5%) PE (5%) Post-cardiotomy shock (11%) CS (16%)	66% flow (5 min) 33% flow (1–2 h) If stable, decannulation If unstable any level, back to full flow	(B) N/A; (H) N/A; (E) LVEF, LVOT VTI, TAPSE, Mitral S'	ECMO removal and survival without MCS for > 24 h	25/38 = 66%
Moury et al. [68]	France; 2018–2019	Prospective cohort; single center; n=15	Post-cardiotomy shock (60%) AMI (40%)	Weaning from ECMO was performed if no onset of a new respiratory, neurologic, or cardiovascular failure was clinically assessed	(B) N/A; (H) N/A; (E) Diaphragm thickening fraction (TF) LVEF	ECMO weaning failure was defined by the death of the patient while being treated with assistance, the need for heart transplantation, and the need for an LVAD	9/15 = 60%
Naruke et al. 2010 [50]	Japan; 1996–2008	Retrospective cohort; single center; n=25	Myocarditis (52%) CS post-AMI (36%) ACHF (12%)	Gradual flow reduction to 1.0 L/min If stable, decannulation	(B) CK, BNP, Creat, CRP; (H) HR, MAP, PAP, PCWP, CVP, CI, ETCO_2_; (E) LVET, LVEF	ECMO weaning	18/25 = 72%
Naruke et al. 2012 [42]	Japan; NR	Cohort; NR; n=30	NR	NR	(B) N/A; (H) SVO_2_, ETCO_2_; (E) N/A	VA-ECMO weaned off without severely deteriorated cardiac output indicated by ETCO2 < 10 mmHg or LVET < 100 ms	19/30 = 63%
North et al. 2018 [46]	USA; 2012–2017	Retrospective cohort; single center; n=60	NR	Gradual flow reduction to 0.5–1.5 L/min (10 min). If stable according to precise criteria’s, decannulation	(B) N/A; (H) MAP, CI, CVP, PA Systolic pressure, PA saturation; (E) LVEF	Successful wean was defined by the following parameters: MAP > 60 mmHg; cardiac index > 2.2 L/min; CVP ≤ 16 mmHg; and EF ≥ 20% on low doses of inotropes or/and pressors followed by decannulation	42/60 = 70%
North et al. 2022 [31]	USA; 2012–2019	Retrospective cohort; single center; n=62	CS post-AMI (100%)	Gradual flow reduction by 0.5–1 L decrement (1–2 min), with echocardiographic evaluation, until 0–0.5 L/min. Decannulation if MAP > 60 mmHg, cardiac index > 2.0, CVP⩽16 mmHg, and LVEF ⩾20% on low-dose inotrope	(B) Troponin I, Creat, CK, AST, ALT, lact; (H) N/A; (E) LVEF	ECMO removal without further mechanical circulatory support defined a successful weaning from ECMO	45/62 = 73%
Omar et al. [33]	USA; 2014–2018	Retrospective cohort; Single center; n=238	Arrhythmia (37%) MI (24%) HF (45%) PE (33%) Post-cardiotomy (25%)	NR	(B) Lact (baseline, 1,3,5, 10 days); (H) N/A; (E) N/A	NR	98/238 = 41%
Oshima et al. [35]	Japan; 1997–2004	Retrospective cohort; single center; n=32	Post-cardiotomy (47%) PE (13%) CS post-AMI (9%) Myocarditis (9%) CMP (3%)	Gradual flow reduction to 1.5–2 L/min If stable, decannulation	(B) Lact; (H) N/A; (E) N/A	ECMO removal and discharged from the ICU	12/32 = 38%
Ouazani et al. [54]	USA; NR	Prospective cohort; single center; n=12	NR	Removal considered when LVEF > 25% and VTI > 10 cm If unstable, weaning trial stopped	(B) N/A; (H) N/A; (E) LVEF, LVOT VTI, TD SaL, SaS, EaL, EaS, LS (LV)	ECMO removal without requiring any further MCS	9/12 = 75%
Pappalardo et al. [9]	Italy; 2008–2013	Observational study; single center; n=42	ECPR (29%) Post-cardiotomy (24%) CS post-AMI (14%) Arrhythmia (13%) PE (4%) Trauma (2%)	Weaning by 0.5 L/min decrement every 6–24 h to 2 L/min If stable, decannulation	(B) BNP, Bili, Creat, CRP; (H) MAP,SBP/DBP, HR, CI, PSP, PDP, PCWP, CVP, SVO_2_; (E) LVEF, LVEDD, LVOT VTI, TR, TD S tricuspid annulus, TAPSE, RVEDD	ECMO removal	49/129 = 38%
Park et al. [17]	South Korea; 2009–2011	Retrospective cohort; single center; n=69	AMI (31.9%) Respiratory failure (18.8%) Sepsis (15.9%) PE (4.3%) Trauma (2.9%)	Gradual flow reduction to 1 L/min/m^2^ If stable, decannulation	(B) ABG, Creat, Hb; (H) SBP, MAP, mean PP; (E) N/A	Survival for 48 h after weaning with mean systolic blood pressure > 90 mmHg	27/69 = 39%
Sawada et al. [40]	Japan; 2013–2017	Retrospective cohort; single center; n=50	CS post-AMI (54%) FM (24%) CMP (10%) other heart disease (12%)	Weaning trial when stable Flow reduction to 1.5–2 L/min Then 0.5–1 L/min If unstable, return to full flow If stable, decannulation	(B) pH, Bic, Lact, Bili, AST,ALT, Creat; (H) PAP, PADP, PCWP, RAP, SVO_2_; (E) LVETc, LVOT VTI, FS, LVEDD/SD	ECMO removal and survival beyond 30 days without needs for further MCS	24/50 = 48%
Sawamura et al. [43]	Japan; 2000–2016	Retrospective cohort; multicenter; n=99	Myocarditis (100%)	NR	(B) CK, BUN, Creat, AST/ALT, LDH, Bili; (H) N/A; (E) LVEF, LVEDD	VA-ECMO decannulation and subsequent discharge	46/99 = 46%
Sugiura et al. [32]	Japan; 2012–2016	Retrospective cohort; multicenter; n=55	CS post-AMI (100%)	Weaning trial when stable Flow reduction to 0.5–1.5 L/min If stable, decannulation	(B) Lact, Creat, Bili; (H) SBP, MAP; (E) LVEF CE-CT LV wall enhancement	ECMO removal	28/55 = 51%
Suhr et al. [34]	Germany; 2006–2017	Retrospective cohort; single center; n=258	NR	NR	(B) Lactate 1, 6, 12, 24 and 36 h; (H) N/A; (E) N/A	NR	136/258 = 53%
Vuthoori et al./Heaney et al. [51]	USA; NR	Prospective cohort; single center; n=34	NR	Weaning trial with close monitoring Flow reduction by 1 L/min decrements If stable, circuit clamped, decannulation	(B) macrophage migration inhibitory factor; (H) CI; (E) LVEF, LV size	ECMO removal and free from pharmacologic and MCS at 30 days post-explant	8/34 = 24%
Wu et al. [47]	Taiwan; 2003–2008	Retrospective cohort; single center; n=72	Post-cardiotomy shock (100%)	Weaning trial when stable Gradual flow reduction to 1 L/min. If stable, decannulation	(B) Creat; (H) MAP, SVO2, MPAP, MAP:MPAP ratio; (E) N/A	ECMO removal	41/72 = 57%
Xu et al. [28]	China; 2019–2021	Retrospective cohort; single center; n=20	Myocarditis (27%) CMP (23%) Ischemic heart disease (21%) ECPR (19%) Other (10%)	Weaning trial when HD stable, signs of cardiac and pulmonary recovery. Flow reduction to 1.5 L/min, If HD stable with low-dose inotrope, PCRTO begins, target—0.5–1 L/min for 30 min with arterial gas, HD and respiratory monitoring. Decannulation if successful	(B) N/A; (H) HR, CVP, PAWP, MAP; (E) LVEDV, LVEF, LVOT VTI, Mitral TD e′ E and lat s′, GLS	Patients who survived for 48 h after withdrawal and did not require ECMO assistance	13/20 = 65%
Yi et al. [41]	China; 2018–2020	Retrospective cohort; single center; n=24	NR	NR	(B) Create, Lact; (H) MAP; (E) LVEF, LVOT VTI, Mitral TD lat s′, GLS	NR	16/24 = 67%
Yoshida et al. [26]	Japan; 2002–2003	Cohort; single center; n=15	CS post-AMI (80%) CPR (20%)	NR	(B) ABG, Lact; (H) HR, MAP, CVP, MPAP, PCWP, SVO_2_, ETCO_2_; (E) N/A	ECMO removal	6/15 = 40%

*ABG* arterial blood gas, *ACHF* acute on chronic heart failure, *AMI* acute myocardial infarction, *ALT* alanine aminotransferase, *AST* aspartate aminotransferase, *BIC* bicarbonate, *BE* base excess, *Bili* bilirubin, *BP* blood pressure, *BNP* brain natriuretic peptide, *BUN* blood urea nitrogen, *CBC* complete blood count, *CI* cardiac index, *CK-MB* creatine kinase MB,* CMP* cardiomyopathy, *CO* cardiac output, *CPR* cardiopulmonary resuscitation, *CREAT* creatinine, CRP c-reactive protein, *CS* cardiogenic shock, *CSt* circumferential strain, *CV* cardiovascular, *CVP* central venous pressure, *DBP* diastolic blood pressure, *ECMO* extracorporeal membranous oxygenation *ETCO*_*2*_ end-tidal CO_*2*_*, e*′ early diastolic peak mitral velocities, *FAC* fractional area change, *FM* fulminant myocarditis, *FS* fractional shortening, *[FWLS]* absolute value of free-wall longitudinal strain, *GLS* global longitudinal strain, *HB* hemoglobin, *HD* hemodynamic, *HI* heterogeneity index, *hTEE* hemodynamic transesophageal echocardiography, *HR* heart rate, *INR* international normalized ratio, *L* liter, *Lact* lactates, *LDH* lactate dehydrogenase, *LS* longitudinal strain, *LV* left ventricle, *LVAD* left ventricular assist device, *LVEDV* left ventricular end-diastolic volume, *LVEF* left ventricular ejection fraction, *LVEDD* left ventricular end-diastolic dimension, *LVESD* left ventricular end-systolic dimension*, LVETc* Left ventricle ejection time corrected, *LVOT VTI* left ventricular outflow tract velocity time integral, *LVPWT* left ventricle posterior wall thickness, *LVWT* left ventricle wall thickness, *MAP* mean arterial pressure, *MAPSE* mitral annular plane systolic excursion, *MCS* mechanical cardiac support, *MFI* microvascular flow index, MPAP mean pulmonary arterial pressure, *MR* mitral regurgitation, *NR* not reported, *PA* pulmonary artery, *PAP* pulmonary artery pressure, *PAPi* pulmonary artery pulsatility index*, PCRTO* pump-controlled retrograde trial off, *PCWP* pulmonary capillary wedge pressure, *PDB* pulmonary diastolic pressure, *PE* pulmonary embolism, *PH* pulmonary hypertension, *PP* pulse pressure, *PPV* percent perfused vessels, *PSP* pulmonary systolic pressure, *PSVD* perfused small vessel density, *PVD* perfused vessel density, *RA* right atrial, *RAP* right atrial pressure, *RV* right ventricle, *RVEDD* right ventricle end-diastolic dimension, *RVEF* right ventricular ejection fraction, *RVSP* right ventricular systolic pressure, *SBF* skin blood flow, *SBP* systolic blood pressure, *S/L* sublingual, *SV* stroke volume, *SVD* small vessel density, *SVO*_*2*_ mixed venous oxygen saturation, *S*′ systolic peak myocardial velocities*, TAPSE* tricuspid annular plane systolic excursion, *TD* tissue Doppler *a’* late diastolic atrial contraction velocities* E* transmitral early peak velocities *Ea/e*′ lateral mitral annulus early diastolic velocities *Sa* lateral mitral annulus peak systolic velocities *Sv* systolic peak velocity, *t-IVT* total isovolumic time, *TNI* troponin I, *TPG* transpulmonary gradient, *TR* tricuspid regurgitation, *VV* veno-venous, *TVD* total vessel density, *V-A-V* veno-arterio-venous, *VTI* velocity time integral, *VVI* velocity vector imaging, *WBC* white blood count

### Quality assessment and risk of bias

The quality assessment of all retained articles was performed by two independent reviewers (FC and EB or OL or KC), with conflicts resolved by consensus by the corresponding author (YAC), using the Cochrane’s Risk of Bias Assessment tool for Non-randomized Studies [21].

## Results

### Study selection and characteristics

**Fig. 2:**
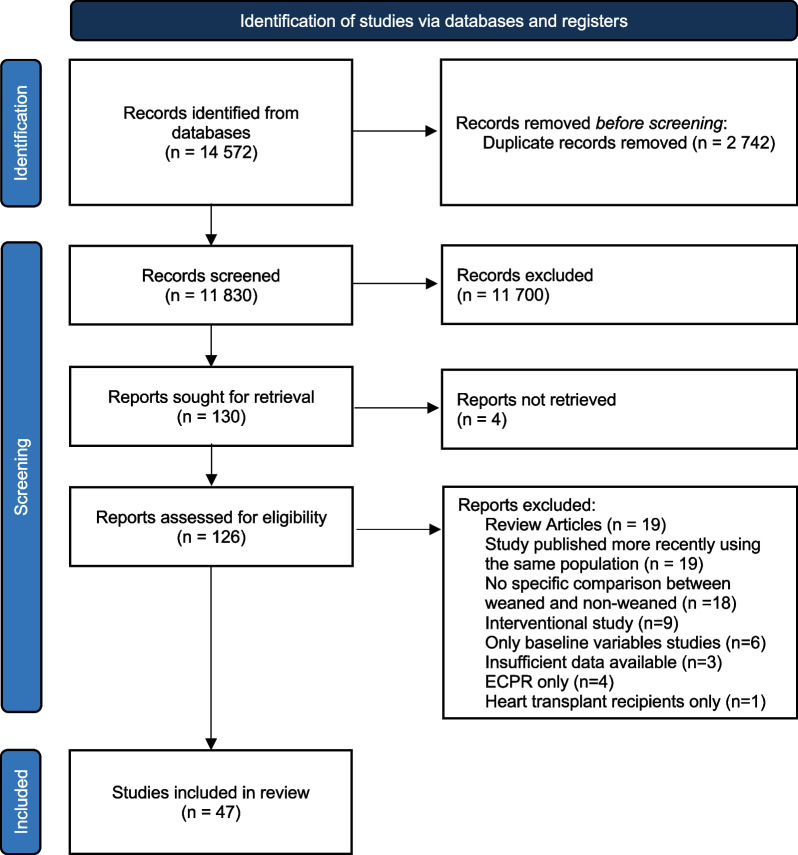
PRISMA flowchart. *ECPR* Extracorporeal cardiopulmonary resuscitation

The search strategy yielded a total of 14,578 records, including 2742 duplicates that were removed. The remaining 11,830 were screened by title and abstract. We reviewed the full text of 130 records to assess eligibility, 47 of which studies were finally selected (Fig. 2 and Table 1). Study sample size ranged from 12 to 258 patients. The main indications for ECMO were cardiogenic shock secondary to acute myocardial infarction (AMI), fulminant myocarditis, other acute cardiomyopathies, post-cardiotomy shock and cardiac arrest. Eight studies included some cardiac arrest patients in their case-mix [9, 22–28]. Specific etiology of cardiac arrest was not specified in most studies. The risk of bias of the included records can be found in Table 2. Fifteen studies were published conference abstracts that did not provide detailed protocols, greatly limiting our methodological assessment. Most studies presented a high risk of bias overall.

**Table 2:**
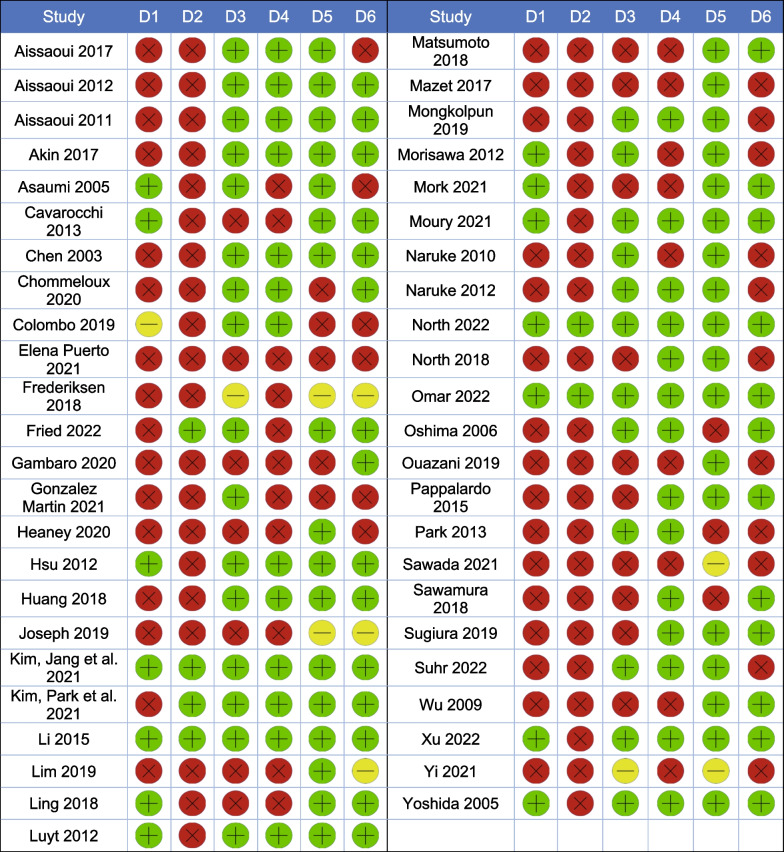
Risk of bias of the included studies

*D1* Selection of participants *D2* Confounding variables *D3* Measurement of exposure *D4* Blinding of outcome assessment *D5* Incomplete outcome data *D6* Selective outcome reporting. *Green* Low risk of bias *Yellow* Unclear risk of bias *Red* High risk of bias

### Main findings: parameters to predict weaning success

Selected records reported on the use of various parameters to predict successful weaning, including biomarkers, microcirculation indices, hemodynamic, respiratory and echocardiographic parameters. Individual study findings are detailed in Table 1 and summarized below. Heterogeneity in methods, timing and conditions of measurements precluded any meta-analysis.

### Biomarkers

#### Cardiac injury

Elevation in biomarkers reflecting the severity of myocardial injury was associated with adverse weaning outcomes in most studies. Lower peak CK-MB was associated with higher weaning success and better initial systolic function in patients with myocarditis or cardiac arrest [5, 29, 30]. Peak CK-MB < 183 U/L predicted weaning success with a sensitivity of 86%, a specificity of 71% and an area under the receiving operator characteristic curve (AUROC) of 0.89 [CI_95_: 0.77–1.00] [30]. Peak troponins were associated with weaning success in AMI patients (*p* = 0.003) [31], but not in a cohort of patients with refractory CS of mixed etiologies where AMI represented only 27% of the cohort [23]. Several other biomarkers including NT-proBNP failed to demonstrate any predictive value for successful weaning [23].

#### Oxygen delivery

Early correction of biomarkers reflecting tissue hypoperfusion appeared to predict successful weaning. Blood lactate levels at 24 h of support were independently associated with successful weaning in patients with cardiac arrest caused by AMI cannulated during or after the arrest (OR 0.52, *p* = 0.018) [32–34]. Lactate clearance in the first 12 h similarly predicted weaning outcomes in post-cardiotomy VA-ECMO (AUROC 0.72; OR 0.3; *p* = 0.023) [16, 34]. However, initial [5, 16, 26, 30, 32, 33, 35–38] and pre-weaning [23, 39–41] lactate values were inconsistently associated with weaning success. We also found conflicting data concerning the association between mixed venous oxygen saturation (SvO_2_) and VA-ECMO weaning success. Hsu et al. found a higher pre-weaning SvO_2_ in patients that survived compared to those that died following weaning [39]. Yoshida et al. found no difference in the mean SvO_2_ while on VA-ECMO in weaned vs non-weaned patients [26]. Finally, Naruke et al. reported that patients with sustained SvO_2_ < 75% during VA-ECMO had a higher rate of weaning success (88 vs 47% *p* < 0.01) compared to patients that experienced periods of SvO_2_ > 75% [42].

#### Organ damage

Similarly, signs of persistent organ damage are adversely linked to weaning outcomes. Higher aspartate aminotransferase at 48 h and 72 h after initiation of ECMO was associated with weaning failure [5, 43].

### Microcirculation

Multiple studies evaluated the use of microcirculatory parameters in VA-ECMO. During the first 48 h of support, successfully weaned patients showed a significantly higher perfused small vessel density (*p* = 0.002), small vessel density (*p* = 0.008) and percent perfused vessels (*p* = 0.02) [44]. Before first weaning attempt, higher skin blood flow (≥ 34 perfusion units) measured by skin laser Doppler was also found to accurately predict weaning success (AUROC = 0.93 [CI_95_: 0.81–1]; sensitivity 83%; and specificity 92%) [36]. During an extracorporeal blood flow (ECBF) reduction trial (50% of initial ECBF), total vessel density and perfused vessel density displayed an AUROC of 0.99 and 0.91, respectively, for the prediction of successful weaning. In two of these studies, microcirculatory indices outperformed commonly used echocardiographic parameters such as left ventricular ejection fraction (LVEF) and left ventricular outflow tract (LVOT) velocity time integral (VTI) [36, 45].

### Macrocirculation

Better early hemodynamic parameters as well as their maintenance during the weaning phase appear to predict weaning success [46]. Higher mean arterial pressure (MAP) and MAP to pulmonary artery pressure ratio at 24 h were associated with successful weaning [32, 47]. Over the first 6 h of extracorporeal support, a pulse pressure (PP) < 30 mmHg, reflecting reduced residual LV ejection, was found to be independently associated with weaning failure (OR: 0.95, Log rank *p* < 0.001) [17]. MAP at time of weaning was independently associated with weaning success (OR: 1.05, *p* = 0.009) [41, 48]. In a cohort of weaned patients, MAP, cardiac index and PP increased significantly from pre-ECMO to weaning despite a reduction in inotropic support. Moreover, compared to patients who died after weaning, patients who survived to ICU discharge had a higher systolic blood pressure (120 [112–140] vs 103 [99–125] mmHg, *p* = 0.04) despite a lower inotropic score [9] and a lower central venous pressure (CVP) [39]. Successfully weaned patients also presented more favorable right ventricular (RV) hemodynamic parameters at 48 and 72 h: lower right atrial to pulmonary capillary wedge pressure (PCWP), lower transpulmonary gradient and higher pulmonary artery pulsatility index [49].

### End-tidal CO_2_

In patients on VA-ECMO, end-tidal CO_2_ (EtCO_2_) is primarily determined by transpulmonary blood flow generated by the native heart. It may thus be used to monitor native cardiac output in this context (Table 3). In a cohort of 37 patients on VA-ECMO, an increase in EtCO_2_ of 5 mmHg or more above previous mean values during two consecutive 12-h periods occurred in all successfully weaned patients and in none of the patients that could not be successfully weaned [50]. This inflection point in EtCO_2_ preceded cardiac index increase. Weaned patients were also found to have a higher absolute EtCO_2_ value at 24 h of extracorporeal support. Their average EtCO_2_ increased from 9 mmHg immediately post-cannulation to 21 mmHg at 24 h (*p* = 0.04) [26].

**Table 3:** Parameters associated with weaning success

Parameters	Definition	Reported performance	Advantages (A)/Disadvantages (D)	References
EtCO_2_	Partial pressure of CO_2_ in the gas mixture at the end of exhaled breath. Under constant ventilator settings, its main determinant is transpulmonary flow from the native heart	Higher values at 24 h in weaned patients; Rapid rise in EtCO2 preceded changes in hemodynamic monitoring and cardiac index; Increase of ≥ 5 mmHg above previous mean values during two consecutive 12-h periods associated with weaning and preceded native cardiac output recovery.	(A) Noninvasive alternative to thermodilution cardiac catheter; (D) Elimination of CO_2_ may depend on pulmonary dead-space, which is increased after CPR, thus reducing ETCO2 level; (D) May vary with change in ventilator settings.	Naruke 2010 [50], Yoshida[26]
LVEF	Relation between the amount of blood expelled during each cardiac cycle relative to the size of the ventricle	Greater increase (> 5%) in the first 48 h and significant improvement from cannulation to weaning associated with weaning; LVEF > 20–25% reported before attempting weaning trial.	(A) Direct marker of systolic function; (D) Load-dependent; (D) Absolute value at weaning inconsistently predicted weaning success.	Aissaoui 2011 [15], Aissaoui 2012 [52], Aissaoui 2017 [55], Akin [45], Asaumi [29], Colombo [22], Gambaro [57], Mazet [24], Ouazani [54], Pappalardo [9], Sawamura [43], Sugiura [32], Vuthoori [51],Yi [41]
LVOT VTI	Velocity time integral (VTI) of a pulsed wave Doppler in the left ventricular outflow tract (LVOT) is directly proportional to the stroke volume of the native heart	Improvement from cannulation to weaning, values above 8.5 cm at weaning associated with success; Threshold of 10 cm reported before attempting weaning trials.	(A) Direct marker of systolic function; (D) Load-dependent.	Aissaoui 2011 [15], Aissaoui 2012 [52], Aissaoui 2017 [55], Colombo [22], Frederiksen [56], Gambaro [57], Gonzalez Martin [27], Lim [48], Mongkolpun [36], Ouazani [54], Sawada [40], Sawamura [43], Yi [41]
TDI	Tissue Doppler velocity imaging (TDI) is a signal which correlates with myocardial motion. TDI is placed on mitral/tricuspid annulus to evaluate longitudinal systolic function	Mitral systolic velocities (Sa) higher in weaned patients both at maximal and minimal (> 6 cm/s) VA-ECMO flow; Any improvement in lateral mitral e' velocity and/or > 10% improvement in tricuspid annular S′ velocity during flow reduction trial is an independent predictor of weaning success.	(A) Load-independent, making it useful to guide the weaning process; (A) Better predictive performance than conventional parameters for weaning success; (D) Angle-dependent for valid measurement, interobserver variability.	Aissaoui 2011 [15], Aissaoui 2012 [52], Frederiksen [56], Mork [25], Ouazani [54], Yi [41]
TAPSE	Tricuspid Annular Plane Systolic Excursion (TAPSE) is a M-mode derived marker of longitudinal right ventricular function	Higher at full flow (15 mm) in succesfully weaned patients.	(D) Limited data; (D) Angle dependency.	Frederiksen [56], Mork [25]
RVEF	Relation between the amount of blood expelled during each cardiac cycle relative to the size of the ventricle	3D derived RVEF > 24.6% at first intent of decannulation associated with weaning; Higher RVEF in patients without ventricular interdependence during weaning trial.	(A) Direct marker of systolic function; (D) Load-dependent; (D) 3D Echo is time consuming and requires offline analysis without immediate assessment; (D) Two-dimensional measurement less reliable	Aissaoui 2017 [55], Huang [61]
Ventricular interdependence (VI)	Phenomenon whereby the function of one ventricle is altered by changes in the filling of the other ventricle	Absence of VI (Dep-) on the last day before weaning predicted successful weaning.	(A) Highlight ventricular response to load variation.	Aissaoui 2017 [55]

### Echocardiographic assessment

#### LV function

Early recovery of LVEF after VA-ECMO initiation has been associated with improved weaning outcomes. LVEF significantly increased after cannulation in weaned patients (from baseline to 24 h: + 8.5%, *p* = 0.012; from 24 to 48 h, + 9.0%, *p* = 0.001) [32]. At 48 h of support, both the absolute LVEF (OR 1.11 [1.01–1.22]; *p* = 0.03) and LVEF change from baseline (OR 1.15 [1.01–1.31]; *p* = 0.03) were independently associated with weaning success [43]. Multiple studies similarly showed a higher LVEF and fractional shortening (FS) [29, 31, 41] as well as lower LV chamber sizes in successfully weaned patients compared to non-weaned patients [39, 51, 52]. Others found LVEF improvement from cannulation to weaning, without significant absolute differences in LVEF values in weaned vs non-weaned patients [9, 22, 53, 54].

LVOT VTI is the most widely used parameter to track LV recovery in patients on VA-ECMO [15, 22, 27, 36, 40, 41, 48, 52, 54–57]. Successfully weaned patients tend to have a higher VTI at the time of weaning, reflecting a better stroke volume. The most commonly reported threshold to predict successful weaning is > 9.5 cm. The threshold itself and the conditions under which it is measured, especially the timing and the ECBF, vary significantly across studies, making comparisons difficult [15, 22, 36, 40, 48, 52, 54–57]. Two studies reported on the accuracy of the LVOT VTI to predict weaning success. Mongkolpun et al. found an AUROC of 0.85 [CI_95_: 0.65–1] [36] and Sawada et al. an AUROC of 0.74, with a sensitivity of 75% and a specificity of 72% at an optimal VTI threshold of > 8.6 cm [40]. The ratio of VTI from cannulation to weaning has also shown a strong association with successful weaning (OR, 2.80, *p* = 0.01) [27, 41, 48]. In two different studies, Aissaoui and colleagues reported a higher VTI in the group of successfully weaned patients compared to those who were not, both at maximal (10.5 vs 6.2 cm, *p* < 0.001) and minimal ECBF during weaning trials (12.8 vs 9.5 cm, *p* = 0.010 and 16.4 vs 8.5 cm, *p* < 0.0001) [15, 52]. The main limitation, Aissaoui found, is the load dependence of most LV function parameters, including LVEF, LVOT VTI, LV systolic velocity, strain and strain rate [52].

Tissue Doppler systolic velocities, which were also found to be higher in successfully weaned patients, are probably less load-dependent [15, 41, 52, 54]. Improvements in lateral e′ velocity and tricuspid annular S′ velocity during a flow reduction trial predicted weaning success with a better performance (AUROC for the presence of both parameters = 0.93 [CI_95:_ 0.863–0.996]; *p* < 0.001) than conventional parameters, such as LVEF > 20–25%, LVOT VTI ≥ 10 cm and mitral annulus S′ ≥ 6 cm/s [58]. Total isovolumic time (t-IVT, Additional file 1: Table S1) improvement in the first 48 h of support was the strongest predictor of successful VA-ECMO weaning in another study [22]. Measured at an ECBF of 1.5 L/min, a corrected LV ejection time (LVETc) to PCWP ratio > 15.9 s/mmHg was a robust predictor of weaning success (AUROC = 0.82; sensitivity = 88%; specificity = 69%) [40].

#### RV function

Improvement in RV function during ECMO also appears to be associated with weaning success [59]. Pappalardo and colleagues observed a decrease in the prevalence of RV failure from 52% pre-ECMO to 36% during weaning in successfully weaned patients [9]. Successfully weaned patients exhibited a higher tricuspid plane systolic excursion at full ECBF (16 vs 8 mm, *p* = 0.02) [25, 56]. Tricuspid lateral annular S′/right ventricular systolic pressure > 0.33 was associated with successful weaning at full and minimal ECMO flow, performing better than conventional LV indexes, reflecting better RV coupling to pulmonary circulation [60]. Huang et al. also found the three-dimensional RV ejection fraction to be the strongest predictor of successful decannulation at first attempt (AUROC 0.90; CI_95_: 0.80–0.99) [61].

#### Biventricular function

In an unblinded study, qualitative echocardiographic assessment of biventricular recovery during a weaning trial had a positive predictive value of 100% [CI_95_: 73–100%] for successful weaning [7]. Interestingly, absence of ventricular interdependence was found to be a robust predictor of successful weaning, with sensitivity of 94% and specificity of 95% [55].

### Weaning trial

Weaning protocols varied significantly across studies, as detailed in Table 1. Our main findings concerning weaning protocols reported in the literature are summarized below.

Criteria used to decide to submit patients to a weaning trial were not always explicitly reported and varied significantly. Most commonly used criteria included a MAP ≥ 60 mmHg and systemic arterial pulsatility on minimal inotropic and vasopressor support [5, 9, 15, 16, 23, 25, 32, 35, 40, 45, 47, 52, 54, 58, 61]. Another commonly stated condition was the resolution of shock as indicated, for instance, by lactate normalization [25, 40, 45, 58, 61], SVO2 > 65% [40, 45] or end-organ dysfunction recovery [7, 16, 40, 44]. Most authors used some form of myocardial improvement, globally [47] or a specific parameter such as LV FS [40], LVEF ≥ 20% and S′ > 6 cm/s [25], LVETc > 200 ms [29, 30, 40], LVOT VTI ≥ 10 cm [25, 56, 57] or TAPSE > 10 mm [25]. Other conditions included the absence of ventricular arrhythmia [47], optimized fluid balance [7, 25, 51] with a low CVP [51, 58, 61] and adequate native lung oxygenation capacity [7, 15, 23, 25, 32, 50, 52, 54] with resolution of pulmonary edema and an inspired oxygen fraction < 50% [7, 25].

In patients who met these criteria, a gradual ECBF reduction was usually advocated with close monitoring of hemodynamic, vasopressor needs and biventricular response to load variation. Even with a minimal ECBF, VA-ECMO still provides significant cardiorespiratory support. As such, some centers reported clamping the circuit in patients who remained stable at 0.5–1.5 L/min of ECBF. Others used a pump-controlled retrograde trial off (PCRTO) to challenge the native heart without the increased risk of thrombus formation associated with circuit clamping [62]. Pump speed was reduced in a controlled fashion until the flow became retrograde (− 0.5 to − 1 L/min). This was maintained for up to an hour, after which the test was considered successful if hemodynamic parameters, vasopressors needs and oxygen requirements remained stable. Ling and colleagues compared the outcomes of seven patients weaned after a successful PCRTO, to 23 patients weaned without PCRTO. The reported number of deaths due to cardiac failure in the PCRTO and conventional groups was 0 and 3, respectively (0 vs 13%, *p* = 0.99) [63]. Lower initial heart rate and PCWP measured by Swan-Ganz catheter at the start of PCRTO were associated with weaning success during this procedure [28].

## Discussion

**Fig. 3:**
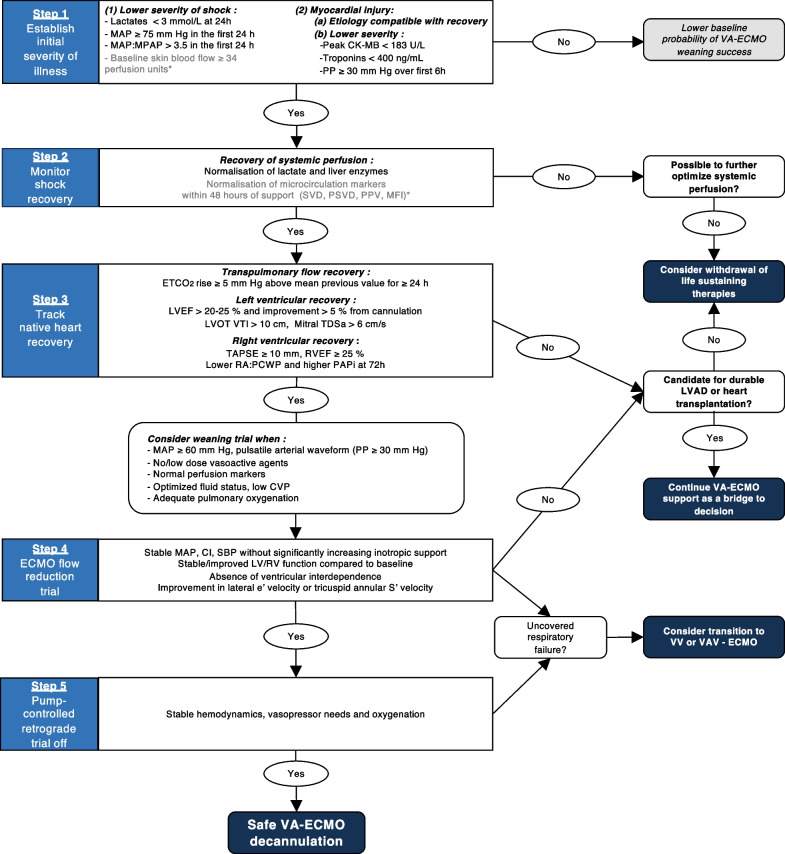
VA-ECMO weaning algorithm. *CK-MB* creatine kinase MB, *CI* cardiac index*, CVP* central venous pressure, *ECMO* extracorporeal membrane oxygenation, *ETCO*_*2*_ end-tidal CO_*2*_*, e*′ early diastolic peak myocardial velocities, *LV* left ventricle, *LVAD* left ventricular assist device, *LVEF* left ventricular ejection fraction, *LVOT VTI* left ventricular outflow tract velocity time integral, *MAP* mean arterial pressure, *MPAP* mean pulmonary arterial pressure, *MFI* microvascular flow index, *PAPi* pulmonary artery pulsatility index*, PCWP* pulmonary capillary wedge pressure, *PH* pulmonary hypertension, *PP* pulse pressure, *PPV* percent perfused vessels, *PSVD* perfused small vessel density, *RA* right atrial, *RV* right ventricle, *RVEF* right ventricular ejection fraction, *SBP* systolic blood pressure, *SVD* small vessel density, *S*′* velocity* systolic peak myocardial velocities*, TAPSE* tricuspid annular plane systolic excursion, *TDSa* systolic tissue Doppler imaging septal mitral annulus, *TPG* transpulmonary gradient, *VV* veno-venous, *V-A-V* veno-arterio-venous, *Investigational markers

In this systematic review of the literature, we found multiple studies reporting on biomarkers, hemodynamic and echocardiographic parameters associated with successful weaning of VA-ECMO in adults. These were mainly small observational studies with a relatively high risk of bias. They nevertheless allowed to draw some important points to guide the weaning process. First, the initial severity of shock and myocardial injury may help establish baseline prognosis. Second, rapid recovery of systemic perfusion is associated with weaning success. Third, signs of native heart recovery are strongly associated with weaning success. Finally, in patients that show signs of native heart recovery, readiness to be weaned can be further assessed with a flow reduction trial, with or without a PCRTO (Fig. 3).

### Proposed evidenced-based algorithm for VA-ECMO weaning

#### Step 1: establishing initial illness severity

Initial severity of illness greatly impacts the odds of being weaned successfully from VA-ECMO. Although this may not directly help determine if a patient is ready to be weaned, it may help set expectations. For instance, a patient with very severe initial myocardial injury and limited organ dysfunction may be considered early for durable left ventricular assist device implantation or heart transplantation. Greater initial myocardial injury, reflected by higher CK-MB, troponins and lower initial systolic function are associated with weaning failure in multiple studies [5, 29–31]. At the time of ECMO initiation, parameters of shock severity such as lactate, end-organ perfusion markers, hemodynamics and extent of myocardial injury predict subsequent weaning and survival. In the first 24 h of support, weaned patients have a higher MAP and PP [17, 32, 47]. Microcirculation indices could also help refine prognostication in the future as they appear to strongly predict weaning outcomes early [36, 44] and independently of hemodynamic parameters, lactates and inotropic support [44, 64, 65].

#### Step 2: monitoring recovery of tissue and organ perfusion

If flow is adequately restored through VA-ECMO, one should expect rapid normalization of markers of hypoperfusion and end-organ damage. Studies showed increased rates of successful weaning with higher lactate clearance and AST normalization in the first 72 h [5, 24, 26, 32–35, 37, 38, 43]. Microcirculatory improvement in the first 48 h has also been associated with successful weaning [36, 44]. Persistence of markers of hypoperfusion should prompt clinicians to try to further improve blood flow by increasing extracorporeal circuit or native heart output, decreasing venous congestion, and seeking local causes of hypoperfusion that could be addressed (such as mesenteric ischemia). In the absence of adequate restoration of tissue perfusion under maximal support, irreversible organ injury usually ensues.

#### Step 3: assessing recovery of native heart function

Once tissue perfusion is restored, the next step is to assess native heart recovery. Under constant minute ventilation, an increase in EtCO_2_ reflects an increase in transpulmonary flow, a reliable marker of native cardiac output recovery that may be observed earlier than changes in hemodynamics [26, 50]. Higher LVOT VTI, both at minimal and maximal ECBF, is a widely used parameter of LV recovery with good predictive performance for weaning success [27, 48]. Weaned patients also tend to display higher LVEF, t-IVT, FS, MAPSE and mitral S′ velocity [25, 29, 40, 43, 53]. Echocardiographic (RV ejection fraction and TAPSE) [25, 59, 61] and hemodynamic (RA/PCWP ratio, TPG and PAPi) parameters of RV function are also strongly associated with weaning success [47, 49]. On full extracorporeal support, LV indices tend to underestimate true LV performance, while RV indices tend to overestimate true RV performance. Tissue Doppler systolic velocities are relatively load-independent [15, 52, 54, 58, 66, 67], making them interesting parameters to follow when patients are still supported with high ECBF.

#### Step 4: performing flow reduction trials

In stable patients with evidence of myocardial recovery, a trial of ECBF reduction is generally attempted prior to decannulation to assess the net hemodynamic effect of reducing the support provided by VA-ECMO. The effect of flow reduction on MAP, CVP and vasopressor-inotropic support is observed. If tolerated, echocardiographic evaluation may be performed. Better indices of LV systolic and diastolic function [7, 15, 51, 52, 55, 57], ventricular interdependence [55] and RV function [58] during flow reduction trials predict favorable weaning outcomes. Importantly, flow reduction may unmask significant underlying hypoxemic respiratory failure that may be associated with increased pulmonary vascular resistance [19].

#### Step 5: pump-controlled retrograde trial off (PCRTO)

Finally, PCRTO may be performed by reducing the centrifugal pump rotation speed until a retrograde flow occurs through the circuit. This creates a controlled arterio-venous fistula, with the blood being pumped through the ECMO circuit by the native heart, returning in the venous system. This significantly challenges the RV which has to accommodate a substantial preload increase. Thereby, it may simulate VA-ECMO decannulation more accurately than flow reduction and may last longer than circuit clamping, which is limited by the risk of clot formation. During retrograde flow, the sweep gas should be turned off to uncover any residual gas exchange impairment [63]. By reversing the flow, there is a theoretical risk of pulmonary embolism through clot detachment from the venous side of the oxygenator. More studies are needed to validate this method.

### Strengths and limitations

This is the first systematic review on VA-ECMO weaning providing an exhaustive and detailed summary of the literature for clinicians and researchers. Our conclusions, however, are limited by the quality of the available literature. We included mostly unblinded observational retrospective studies with small sample sizes and a high risk of bias. Many of the reported parameters, including ETCO_2_, were studied in small cohorts of patients. This warrants cautious interpretation about their role in clinical practice. Also, the microcirculatory parameters are still in the investigative stage and deserve more validation before drawing conclusions that will influence VA-ECMO weaning guidance. Variability in weaning protocols and in the definition of successful weaning make interpretation and comparison between studies difficult. Most studies did not provide formal assessments of predictive accuracy of the proposed indices and only reported on associations. Selection bias was often present as patients who were not considered for weaning because of complications or futility were often not included. There was often significant residual confounding. These limitations underline the need for high-quality research in this area, more robust data on investigational markers and standardization of weaning protocols and successful VA-ECMO weaning definition.

## Conclusion

We found a large number of studies, of generally low quality, reporting on parameters associated with successful weaning of VA-ECMO in adults. We propose a stepwise approach to weaning based on our findings. First, the initial severity of shock and myocardial injury may help establish baseline prognosis. Then, signs of reversal of tissue hypoperfusion and native heart recovery may help determine readiness to be weaned. Finally, careful assessment of the physiological response to extracorporeal blood flow reduction and/or reversal may allow identification of patients who are ready to be safely decannulated.

## Supplementary Information

**Additional file 1.** Supplementary tables.

## Data Availability

All data generated or analyzed during this study are included in this published article and its supplementary information file.

## Abbreviations

ABG: Arterial blood gas
ACHF: Acute on chronic heart failure
AMI: Acute myocardial infarction
ALT: Alanine aminotransferase
AST: Aspartate aminotransferase
AUROC: Area under the receiving operator characteristic curve
BIC: Bicarbonate
BE: Base excess
Bili: Bilirubin
BP: Blood pressure
BNP: Brain natriuretic peptide
BUN: Blood urea nitrogen
CBC: Complete blood count
CE-CT: Contrast enhanced computed tomodensitometry
CI: Cardiac index
CK-MB: Creatine kinase MB
CI_95_: 95% confidence interval
CMP: Cardiomyopathy
CO: Cardiac output
CREAT: Creatinine
CS: Cardiogenic shock
CSt: Circumferential strain
CRP: C-reactive protein
CPR: Cardiopulmonary resuscitation
CV: Cardiovascular
CVP: Central venous pressure
DBP: Diastolic blood pressure
ECBF: Extracorporeal blood flow
ECLS: Extracorporeal life support
ECMO: Extracorporeal membranous oxygenation
ECPR: Extracorporeal cardiopulmonary resuscitation
ELSO: Extracorporeal Life Support Organization
ETCO_2_: End-tidal CO_*2*_
e′: Early diastolic peak mitral velocities
FAC: Fractional area change
FM: Fulminant myocarditis
FS: Fractional shortening
[FWLS]: Absolute value of free-wall longitudinal strain
GLS: Global longitudinal strain
HB: Hemoglobin
HD: Hemodynamic
HI: Heterogeneity index
hTEE: Hemodynamic transesophageal echocardiography
HR: Heart rate
INR: International normalized ratio
L: Liter
Lact: Lactates
LDH: Lactate dehydrogenase
LS: Longitudinal strain
LV: Left ventricle
LVAD: Left ventricular assist device
LVEDV: Left ventricular end-diastolic volume
LVEF: Left ventricular ejection fraction
LVEDD: Left ventricular end-diastolic volume
LVESD: Left ventricular end systolic volume
LVETc: Left ventricle ejection time corrected
LVOT VTI: Left ventricular outflow tract velocity time integral
LVPWT: Left ventricle posterior wall thickness
LVWT: Left ventricle wall thickness
MAP: Mean arterial pressure
MAPSE: Mitral annular plane systolic excursion
MCS: Mechanical cardiac support
MFI: Microvascular flow index
MPAP: Mean pulmonary arterial pressure
MR: Mitral regurgitation
NR: Not reported
PA: Pulmonary artery
PAP: Pulmonary artery pressure
PAPi: Pulmonary artery pulsatility index
PCRTO: Pump-controlled retrograde trial off
PCWP: Pulmonary capillary wedge pressure
PDB: Pulmonary diastolic pressure
PE: Pulmonary embolism
PH: Pulmonary hypertension
PP: Pulse pressure
PPV: Percent perfused vessels
PSP: Pulmonary systolic pressure
PSVD: Perfused small vessel density
PVD: Perfused vessel density
RA: Right atrial
RAP: Right atrial pressure
RV: Right ventricle
RVEDD: Right ventricle end-diastolic volume
RVEF: Right ventricular ejection fraction
RVSP: Right ventricular systolic pressure
SBF: Skin blood flow
SBP: Systolic blood pressure
S/L: Sublingual
SV: Stroke volume
SVD: Small vessel density
SVO_2_: Mixed venous oxygen saturation
S′: Systolic peak myocardial velocities
TAPSE: Tricuspid annular plane systolic excursion
TD: Tissue Doppler
a′: Late diastolic atrial contraction velocities
E: Transmitral early peak velocities
Ea/e′: Lateral mitral annulus early diastolic velocities
Sa: Lateral mitral annulus peak systolic velocities
Sv: Systolic peak velocity
t-IVT: Total isovolumic time
TNI: Troponin I
TPG: Transpulmonary gradient
TR: Tricuspid regurgitation
VV: Veno-venous
TVD: Total vessel density
V-A-V: Veno-arterio-venous
VAD: Ventricular assist device
VTI: Velocity time integral
VVI: Velocity vector imaging
WBC: White blood count
