# Supplementary material for: Parameters associated with successful weaning of veno-arterial extracorporeal membrane oxygenation: a systematic review
Source: Crit Care. 2022 Dec 5;26:375. doi: 10.1186/s13054-022-04249-w (PMC9724323; doi:10.1186/s13054-022-04249-w)
Supplement: Supplementary file 1 — Additional file 1. Supplementary tables. [file 13054_2022_4249_MOESM1_ESM.docx]

| **Parameters**  **Appendix**  **Supplementary table 1 – Definitions of reported markers** | **Definition / Physiology** |
| --- | --- |
| Macrophage migration inhibitory factor | MIF possesses several biological activities directed toward proinflammatory phenotype, stimulated by glucocorticoids but not supressed by their anti-inflammatory properties. |
| Microcirculation | Assessed based on flow in arterioles, capillaries, and venules as well as the density of this network.  Since the microcirculation is the primary site of oxygen and nutrient exchange, therapeutic interventions aimed at increasing organ perfusion in cardiogenic shock should be accompanied by improved microvascular perfusion. |
| Right atrial : Pulmonary Capillary Wedge Pressure (RA : PCWP) ratio | The ratio of right atrial pressure to pulmonary capillary wedge pressure. Higher ratios suggest RV failure as the cause of high right atrial pressure. Conversely, a lower ratio points toward LV systolic/diastolic failure. |
| Transpulmonary gradient (TPG) | It is calculated by subtraction of mean pulmonary artery pressure (MPAP) – PCWP. |
| Pulmonary artery pulsatility index (PAPi) | It is calculated as the pulmonary artery pulse pressure (PAPP) divided by the right atrial pressure (RAP). It is an indicator of right heart function. Pulmonary artery systolic pressure (PASP) is seen as an indirect indicator of RV contractile function and high RAP as an indicator of a failing ventricle. |
| Central venous pressure (CVP) | Pressure measured in the vena cava, before its junction with right atrium. It can be used as an estimation of preload and right atrial pressure. |
| Total isovolumic time (t-IVT) | It represents the sum of total isovolumetric contraction and relaxation times. It is a sensitive marker of electromechanical efficiency and systolic-diastolic interaction. |
| Left ventricular ejection time (LVET) | It measures the period of blood flow across the aortic valve. When corrected for heart rate (LVETc), it is influenced by load conditions and contractile state. |
| Right ventricular (RV) – Pulmonary circulation (PC) coupling | Different parameters studied :   - Tricuspid annular S’ / RVSP - RV FAC / RVSP - TAPSE / RVSP - RV FWLS / RVSP   These parameters were validated as non-invasive surrogate of RV – arterial coupling in the context of pulmonary hypertension. They may reflect the RV load adaptability. |
| Diaphragm thickening fraction | Diaphragm thickening fraction represents the relative inspiratory increase in thickness of the diaphragm at the zone of apposition with the chest wall. It may be used a surrogate to quantify work of breathing. |

**Supplementary table 2 – Search strategy**

| **Ovid MEDLINE(R) ALL ; database inception to April 10^th^ 2022** | |
| --- | --- |
| 1 | Extracorporeal Membrane Oxygenation/ or Cardiopulmonary Bypass/ or Heart-Lung Machine/ or Extracorporeal Circulation/ or cardiopulmonary resuscitation/ |
| 2 | (ECMO or extracorporeal membrane oxygenation or extra-corporeal membrane oxygenation or VA-ECMO or VAECMO or Extracorporeal Life Support? or Extra-corporeal Life Support? or ECLS or ECLS treatment? or extracorporeal circulation? or extra-corporeal circulation? or venoarterial extracorporeal membrane oxygenation? or veno arterial extracorporeal membrane oxygenation? or venoarterial extra-corporeal membrane oxygenation? or veno arterial extra-corporeal membrane oxygenation? or Venoarterial extracorporeal life support? or Veno arterial extracorporeal life support? or Venoarterial extra-corporeal life support? or Veno arterial extra-corporeal life support? or venoarterial ECMO or veno arterial ECMO or ECMO treatment? or (ECMO adj2 (cpr or Cardiopulmonary resuscitation)) or ECPR or extracorporeal cardiopulmonary resuscitation or CPS or Cardiopulmonary Support).tw. |
| 3 | 1 or 2 or (resuscitation/ and (extracorporeal or extra-corporeal).tw.) |
| 4 | Withholding Treatment/ or Ventilator Weaning/ or Device Removal/ |
| 5 | (taper$4 or wean* or treatment? withdrawal or withdraw$4 treatment? or treatment cessation or (cessation adj2 treatment?) or device removal? or success or weaning success or (succes* adj3 weaning) or successful or decannulation or (decannulation? adj3 success) or (success* adj3 decannulation?) or termination or trial off or (wean$3 adj3 trial?) or ((timing or strateg$3) adj4 weaning?)).tw. |
| 6 | 4 or 5 |
| 7 | 3 and 6 |
| 8 | 7 not exp pediatrics/ not exp adolescent/ not exp infant/ not exp child/ not accidents, traffic/ not (venovenous or veno-venous or vv or transplant$6).ti. not ((Animals/ or Models, Animal/ or Disease Models, Animal/) not Humans/) not ((animal or animals or canine* or dog or dogs or feline or hamster* or lamb or lambs or mice or monkey or monkeys or mouse or murine or pig or pigs or piglet* or porcine or primate* orrabbit* or rats or rat or rodent* or sheep* or veterinar*) not (human* or patient*)).ti,kf,jw. |
| 9 | 7 not 8 |

| **Google Scholar – database inception to April 10^th^ 2022** |
| --- |
| (ECMO OR extracorporeal membrane oxygenation OR VA-ECMO OR Extracorporeal Life Support OR ECLS OR extracorporeal circulation) (tapering OR weaning OR withdrawing OR device removal OR successful weaning OR successful decannulation OR weaning trial) |

| **Embase : database inception to April 10^th^ 2022** | |
| --- | --- |
| 1 | exp extracorporeal oxygenation/ or exp extracorporeal circulation/ or heart lung machine/ |
| 2 | (ECMO or extracorporeal membrane oxygenation or extra-corporeal membrane oxygenation or VA-ECMO or VAECMO or Extracorporeal Life Support? or Extra-corporeal Life Support? or ECLS or ECLS treatment? or extracorporeal circulation? or extra-corporeal circulation? or venoarterial extracorporeal membrane oxygenation? or veno arterial extracorporeal membrane oxygenation? or venoarterial extra-corporeal membrane oxygenation? or veno arterial extra-corporeal membrane oxygenation? or Venoarterial extracorporeal life support? or Veno arterial extracorporeal life support? or Venoarterial extra-corporeal life support? or Veno arterial extra-corporeal life support? or venoarterial ECMO or veno arterial ECMO or ECMO treatment? or (ECMO adj2 (cpr or Cardiopulmonary resuscitation)) or ECPR or extracorporeal cardiopulmonary resuscitation or CPS or Cardiopulmonary Support).tw. |
| 3 | 1 or 2 or (resuscitation/ and (extracorporeal or extra-corporeal).tw.) |
| 4 | treatment withdrawal/ or weaning/ or device removal/ |
| 5 | (taper$4 or wean* or treatment? withdrawal or withdraw$4 treatment? or treatment cessation or (cessation adj2 treatment?) or device removal? or success or weaning success or (succes* adj3 weaning) or successful or decannulation or (decannulation? adj3 success) or (success* adj3 decannulation?) or termination or trial off or (wean$3 adj3 trial?) or ((timing or strateg$3) adj4 weaning?)).tw. |
| 6 | 4 or 5 |
| 7 | 3 and 6 |
| 8 | 7 not exp pediatrics/ not exp child/ not exp infant/ not exp adolescent/ not medical society/ not audiovisual equipment/ not ((exp animal/ or exp juvenile animal/ or adult animal/ or animal cell/ or animal tissue/ or nonhuman/ or animal experiment/ or animal model/) not human/) not (veno-venous or vv or venovenous or transplant$6 or fibrinogen? or endocarditis or rejection? or pediat$6 or child$4 or teen$5 or neonat$3 or infant? or prematur$5 or fetus).ti. |
| 9 | 7 not 8 |

| **ProQuest – database inception to April 10^th^ 2022** |
| --- |
| (ECMO or extracorporeal membrane oxygenation or extra-corporeal membrane oxygenation or VA-ECMO or VAECMO or Extracorporeal Life Support* or Extra-corporeal Life Support* or ECLS or ECLS treatment* or extracorporeal circulation* or extra-corporeal circulation* or venoarterial extracorporeal membrane oxygenation* or veno arterial extracorporeal membrane oxygenation* or venoarterial extra-corporeal membrane oxygenation* or veno arterial extra-corporeal membrane oxygenation* or Venoarterial extracorporeal life support* or Veno arterial extracorporeal life support* or Venoarterial extra-corporeal life support* or Veno arterial extra-corporeal life support*or venoarterial ECMO or veno arterial ECMO or ECMO treatment* or ECMO NEAR/2 cpr or ECMO NEAR/2 Cardiopulmonary resuscitation or ECPR or extracorporeal cardiopulmonary resuscitation or Cardiopulmonary Support).partout sauf Texte intégral AND (taper* or wean* or treatment* withdrawal or withdraw* treatment* or treatment cessation or cessation NEAR/2 treatment* or device removal* or success or weaning success or succes* NEAR/3 weaning or decannulation or decannulation* NEAR/3 success or success* NEAR/3 decannulation* or termination or trial off or wean* NEAR/3 trial* or timing NEAR/4 weaning* or strateg* NEAR/4 weaning*).partout sauf texte intégral NOT (pediatrics OR infant OR adolescent OR neonat* OR animal*). TI |

| **OpenGrey – database inception to April 10^th^ 2022** |
| --- |
| (ECMO OR extracorporeal membrane oxygenation OR ECLS OR extracorporeal life support OR Assistance circulatoire extra corporelle OR Assistance circulatoire extra-corporelle OR Assistance circulatoire extracorporelle OR oxygénateur extracorporel à membrane OR oxygénation extracorporelle) and (weaning OR sevrage OR decannulation)  (Assistance circulatoire extracorporelle OR oxygénateur extracorporel à membrane OR oxygénation extracorporelle) |
